# Laparoscopic repair of ventral / incisional hernias

**DOI:** 10.4103/0972-9941.27737

**Published:** 2006-09

**Authors:** Pradeep K Chowbey, Anil Sharma, Magan Mehrotra, Rajesh Khullar, Vandana Soni, Manish Baijal

**Affiliations:** Minimal Access and Bariatric Surgery Centre, Sir Ganga Ram Hospital, New Delhi - 110 060, India

**Keywords:** Laparoscopic ventral hernia repair, Incisional hernia

## Abstract

Despite its significant prevalence, there is little in the way of evidence-based guidelines regarding the timing and method of repair of incisional hernias. To add to the above is the formidable rate of recurrence that has been seen with conventional tissue repairs of these hernias. With introduction of different prosthetic materials and laparoscopic technique, it was hoped that an improvement in the recurrence and complication rates would be realized. The increasing application of the laparoscopic technique across the world indicates that these goals might indeed be achieved.

## INCIDENCE AND PRESENTATION

Incisional hernia is a potential complication of any laparotomy or laparoscopic procedure. Prospective studies have reported an incidence of between 7.4 and 11%.[1–4] Abdominal wall defects occur within the first 5 years after the surgical incision is made, but many develop long afterward. Estimated 65% of hernias develop in the first 5 years after surgery and one in three hernias cause symptoms as shown in a larger prospective study.[3] The late-appearing hernias were smaller than the early ones and caused little trouble. In another large prospective study, the actuarial rate of incisional hernia was shown to be 13% at 5 years, occurring during the first 24 months in 80% of cases.[2]

## RISK FACTORS

The risk factors for recurrence include factors related to patient's status, underlying disease, surgical technique and postoperative complications. Surgical technique of wound closure also plays a role. Modified Smead Jones technique[5] (interrupted closure of the abdominal wall using nonabsorbable suture material, with sutures taken in a ‘far near-near far’ fashion) has been shown to decrease the incidence of early wound dehiscence. Perioperative factors appear to have the most significant correlation to incisional hernia formation, with wound infection being the most consistently reported risk factor. Other perioperative factors include deep abscesses, perioperative gastrointestinal complications and early reoperations.[2,6]

It is essential to understand that incisional hernias generally manifest after considerable delay following the initial surgery. The incidence of hernia development shows a linear curve and therefore there is much more than the technique of wound closure that contributes to the formation of these hernias. For example, patients with an aortic aneurysm or a proven defect of collagen synthesis exhibit an increased incidence of incisional hernias and thus require more extensive reinforcement. The other important aspect, especially in the repair of recurrent hernias, is that repetition of a previously inadequate technique frequently fails.[7]

## CONVENTIONAL REPAIRS FOR INCISIONAL / VENTRAL HERNIA

Recurrence rates of 50% after suture repair of an incisional hernia were observed in several studies. It was the introduction of mesh by Usher *et al.* in 1958 that opened a new era.[8,9] Examination of the literature shows that the results are independent of mesh type and operative technique. Reinforcement of the closed hernial gap by mesh is based on the concept of ingrowth of fibrous tissue into prosthetic material, forming a mesh-scar compound.

In summary, the use of mesh can reduce the recurrence rate from 40–50% to about 10%.[10]

## RATIONALE FOR LAPAROSCOPIC REPAIR

In the laparoscopic technique, the mesh is placed in an intraperitoneal location and less often in the preperitoneal location, where the rise in the intra-abdominal pressures is totally diffused along each square inch of the mesh and not along a tenuous suture line, as happens in conventional suture repairs. An increase in the intra-abdominal pressures thus helps to keep the mesh in place rather than displace it, as is the case in conventional overlay repairs. Therefore, as with the retromuscular, sublay repair described by Stoppa,[11] Rives *et al*[12] and Wantz,[13] the laparoscopic repair of ventral defects capitalizes on the physics of Pascal's principle of hydrostatics by using the forces that create the hernia defect to hold the mesh in place.

The laparoscopic approach affords the surgeon the ability to clearly and definitively define the margins of the hernia defect and to identify additional defects that may not have been clinically apparent preoperatively. Complete visualization of the fascia underlying the previous incision allows for identification of smaller ‘Swiss-cheese’ defects that could be missed in an open approach.[14] One of the key determinants to a high recurrence rate following conventional repairs is the phenomenon of occult hernias. These are the hernias liable to be missed during an open repair. The occult hernia may either be in relation to the primary hernia or at a distance from the primary hernia but within the previous scar or it may be a hernia totally unrelated to the previous scar. The advantage of laparoscopic approach is that not only the primary hernia but the entire scar and not only the scar but the entire abdominal wall can be inspected. Such an approach ensures that occult hernias are detected and treated.

Finally, it stands to reason that a wide overlap of the defect with mesh would help to prevent the intra-abdominal forces from displacing the mesh into the defect. The laparoscopic approach allows for easier placement of a larger prosthesis with good overlap. In the open approach, attaining an overlap of 3 to 5 cm requires extensive soft tissue dissection, with resultant increase in wound complications. This advantage is more prominent in obese patients and those with larger defects.

## CONTRAINDICATIONS TO LAPAROSCOPIC REPAIR OF INCISIONAL / VENTRAL HERNIA

The laparoscopic repair is contraindicated in patients in whom a safe intraperitoneal access cannot be obtained, as in patients with multiple scars on the abdominal wall. The laparoscopic repair should not be attempted in patients with large defects where a 3 to 5 cm overlap of the mesh is not possible intra-abdominally. Also patients with a large amount of redundant skin and fat on the abdominal wall are better suited for an abdominoplasty procedure. The other relative contraindications are poor cardiovascular or respiratory reserve. Bleeding disorders and coagulation defects are also contraindications, as for any other surgery.

## TECHNIQUE

The technique of laparoscopic repair of incisional hernias involves intraperitoneal onlay mesh placement and in selected cases, partial or total extraperitoneal mesh placement. Like most surgical procedures, there are numerous variations described in literature, but surgical principles are constant.

### Initial access

This may include Veress needle - closed method; Hasson's technique - open method; or use of an optical trocar. More often, a Veress needle entry is possible without additional risk; however, in cases of severely scarred abdomen - ‘battlefield abdomen’[14] - an open entry is the method of choice. Before commencement of the operation, it should be ensured that an orogastric tube is placed *in situ* to decompress the stomach and also a urinary catheter if the hernia is located in the lower abdomen. The most preferred site for initial access is the Palmer's point - a point 3 cm below the left costal margin in the midclavicular line; one is least likely to encounter intra-abdominal adhesions at this point.[15] Alternative sites include the right hypochondrium and the right and left iliac fossae.

### Port placement - number and size

Generally three trocars are adequate for small to moderate size hernias; however, if required there should be no hesitation to place more trocars. Trocars are placed such that all the possible defects may be explored and reduction of contents performed. Ports are placed in the form of an arc around the primary hernial defect (triangulation of trocars). At least one 10/12 mm trocar is required for insertion of the mesh.

### Adhesiolysis and reduction of sac contents

Once the appropriate number of trocars is introduced into the abdomen at adequate locations, adhesiolysis is commenced. Adhesions of the abdominal contents to the hernial sac and the surrounding abdominal wall are lysed and the contents of the hernia are reduced. In most patients, the omentum forms the content of the hernial sac; and in less than one-third of patients, the bowel forms a content of the hernial sac. Adhesiolysis is preferably performed using cold scissors or with the help of any of the available energy sources.

### Mesh sizing and placement

**Figure 1:**
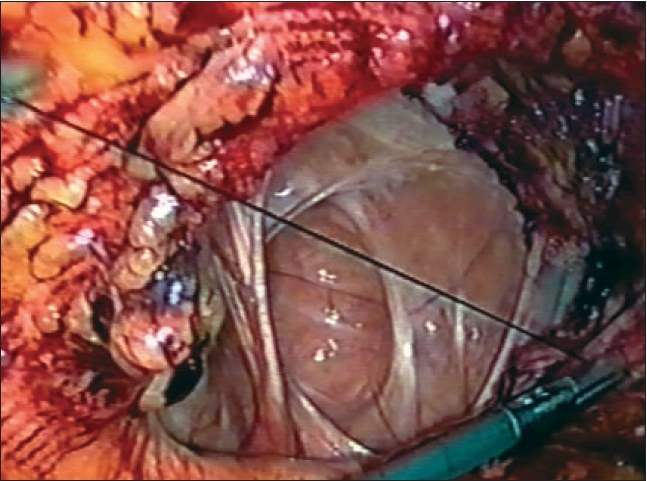
Laparoscopic view of the recurrent incisional hernia following open mesh repair

After adequate delineation of all the defects [Figure 1] and the scar tissue, the size of the mesh required is assessed. This is done by desufflation of the abdomen after marking the defect on the skin by direct palpation of the defects under laparoscopic guidance. To the measured size of the defect, 3 to 5 cm is added in all directions to provide for overlap in all directions. The mesh configuration is also marked on the abdominal wall and the site for four transfascial sutures is marked. All necessary precautions are taken to avoid contamination of the mesh with skin pathogens, by avoiding contact of the mesh with the abdomen of the patient. The mesh is then rolled and inserted through a trocar of adequate caliber (at least 10/12 mm). This may need enlargement of an existing trocar to up to 18 mm size.

### Fixation of mesh

**Figure 2:**
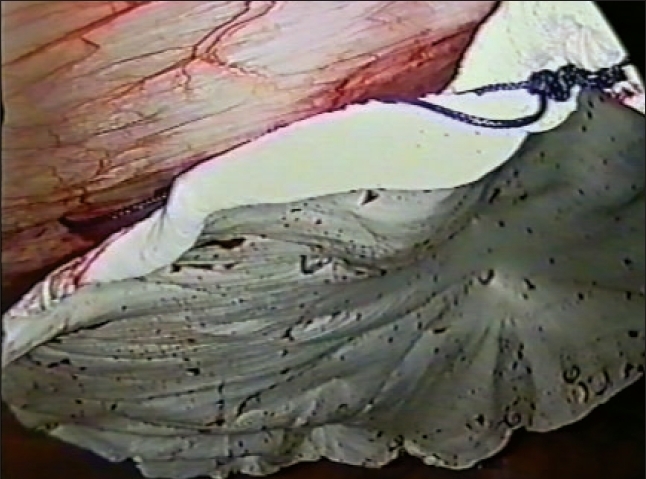
Laparoscopic view of ePTFE mesh after intra-abdominal onlay placement

Transfascial sutures are taken at the previously marked sites on the abdominal wall through a stab incision with the help of a fascia closure needle. The mesh is additionally fixed by means of a fixation device. A ‘double crown’ technique is used, in which the inner layer of fixation devices [Table 1] is applied around the defect and the outer one at the periphery of the mesh, to reduce the chances of recurrence [Figure 2].

**Table 1:** Review of available fixation devices

Material used	Product	Manufacturer
Titanium staple	EMS stapler^®^	Ethicon Endo-Surgery, Inc. Cincinnati, OH 45242
Titanium staple	Universal stapler^®^	AutoSuture/US surgical, Norwalk, CT, USA
Titanium tack	ProTack^®^	Tyco/US surgical, Norwalk, CT, USA
Stainless steel	Salute^®^	CR Bard, Inc, Murray Hill, New Jersey, USA
Nitinol anchor	EndoAnchor^®^	Ethicon Endo-Surgery, Inc. Cincinnati, OH 45242
Absorbable polylactic acid, I - clip	Sofradim pariefix^®^	Sofradim corp, Floreane Medical Implants, USA

Finally the omentum is laid over the underlying bowel loops to prevent direct contact with the mesh. Ports larger then 10 mm are closed under vision using a fascia closure needle. Port closure is particularly important in these patients as they have a tendency to hernia formation.

Fixation of all the biomaterials to the abdominal wall is required until sufficient ingrowth has made collagen impregnation sufficiently strong to ensure repair of the fascial defect.

The physics of mesh fixation during laparoscopic ventral hernia repair does not support the sole placement of tacks/other fixation devices. The majority of the meshes used for laparoscopic ventral hernia repair are roughly 1 mm thick and the spiral tacks are 4 mm long and take up a 1 mm profile on the surface of the patch. A perfectly placed tack can be expected to penetrate only 2 mm beyond the mesh; hence tacks will likely not give the same holding strength as a full thickness abdominal wall suture. Because many candidates presenting for laparoscopic ventral hernia repair are obese (having a substantial amount of preperitoneal fat), the 2 mm penetration of the tack will not reach the fascia in most cases. Experimental studies have confirmed the superior strength of sutures versus tacks alone in mesh fixation to the abdominal wall. They have concluded that suture fixation of the mesh in laparoscopic ventral hernia repair is imperative, especially during the early period of mesh incorporation.[16,17]

### Choice of biomaterial

The biomaterials (meshes) available to repair these hernias laparoscopically have undergone many changes over the last several years. In fact, there are new products that have been recently introduced and several more are in developmental stages [Table 2]. All seek to achieve two goals: rapid and permanent ingrowth into the prosthesis and diminution of the risk of intestinal adhesions. There are two types of such biomaterials: synthetic and collagen based.

**Table 2:** Brief review of available synthetic biomaterials

Biomaterial	Product	Manufacturer
Expanded Polytetrafloroethylene (ePTFE)	DualMesh^®^	WL Gore and Associates, Flagstaff, AZ, USA.
Polypropylene and ePTFE	Composix^®^	CR Bard, Cranston, NJ, USA.
Polyester and collagen	Parietex^®^	Sofradim, Villefranche-sur-Saône, France.
Polypropylene and collagen	Parietene^®^	Sofradim, Villefranche-sur-Saône, France.
Polypropylene	Prolene^®^	Ethicon, Somerville, NJ, USA.
Polypropropylene, polydioxanone and oxidized regenerated cellulose	Proceed^®^	Ethicon, Somerville, NJ, USA.

### Collagen-based biomaterials

The collagen-based biomaterials represent relatively new additions for the laparoscopic surgeon. All have been treated to eliminate all cells and proteins other than collagen that might evoke adverse reactions. All of them have had limited use in laparoscopic repair of incisional hernias but may be of benefit when used in an infected field.

## RESULTS

In incisional hernia repair, the long-term results are probably more important than the short-term results. The long-term results are now emerging as the technique has been performed in many centers, now, for more than a decade. There is more data also available now as compared to the past due to the increasing popularity of the technique.

### Recurrence [Table 3]

**Table 3:** Recurrence rates with laparoscopic repair of incisional / ventral hernias

Name	Year	*N*	Mesh overlap	Recurrence (%)	Mean *f/u*
Franklin[19]	2004	384	3-5 cm	11 (2.9)	47.1 mths
LeBlanc[20]	2003	200	≥3 cm	13 (6.5)	36 mths
Heniford[21]	2003	850	≥ 3–5 cm	40 (4.7)	20.2 mths
Carbajo[22]	2003	270	5 cm	12 (4.4)	44 mths
Bageacu[23]	2002	159	5 cm	19 (11.9)	49 mths
Berger[24]	2002	150	3–5 cm	4 (3.0)	15 mths
Chowbey[25]	2000	202	3–5 cm	3 (1.6)	35 mths
Toy[26]	1998	144	-	6 (4.4)	7.4 mths
Total -		2359		108 (4.57)	

Mechanisms of recurrence of ventral hernia described in the literature, in decreasing order of frequency are infection, lateral detachment of the mesh, inadequate mesh fixation, inadequate mesh, inadequate overlap, missed hernias, increased intra-abdominal pressure and trauma.[18]

### Mesh infection

Mesh infection remains a serious complication with laparoscopic ventral hernia repair. Although the incidence is very low, the consequences are severe. Skin pathogens are responsible for most mesh infections. Every effort is made to avoid contact of the mesh with the abdomen.

Infection of the polypropylene mesh can be managed locally with surgical drainage and excision of exposed, unincorporated segments. Meshes containing ePTFE require removal of the prosthetic material in most cases.

### Seroma formation

Seroma formation is not unique to the laparoscopic approach. Most seromas develop above the mesh and within the retained hernial sac. The rate of seroma formation in reported series varies depending on when investigators evaluated it. The mean incidence of seroma at 4 to 8 weeks is 11.4% in large reported series. In the largest multi-institutional trial, seromas that were clinically apparent more than 8 weeks postoperatively were considered a complication and occurred in 2.6% cases.[20] Regardless of whether they are aspirated under sterile conditions or allowed to resolve, seromas rarely result in long-term problems. Aspiration is recommended for seromas that enlarge or persist before they reach large sizes, when rarely they can give rise to necrosis of the overlying skin. The patients should be counseled preoperatively regarding the possibility of seroma formation after laparoscopic repair.

### Chronic pain

After laparoscopic ventral hernia repair, patients will occasionally complain of persistent pain and point tenderness at a transabdominal suture site. Transabdominal suture site pain after laparoscopic ventral hernia repair is not uncommon and occurs in 1 to 3% of patients in the reported series of such repairs.[18–20]

The discomfort at the transabdominal fixation suture sites typically resolves within 6 to 8 weeks. A possible explanation for the pain may be that the transabdominal suture entraps an intercostal nerve as it courses through the abdominal muscles. Local muscle ischemia is another possibility. Treatment includes oral NSAIDs and injection of local anesthetic agent at the painful suture sites. Most of those responding to therapy require only one injection. This is perhaps due to the ability to block the afferent signal temporarily and allow the hypersensitivity to subside.[27]

### Postoperative morbidity

Causes of postoperative morbidity, apart from those mentioned above, are unrecognized enterotomy, wound infection, intraperitoneal abscess and respiratory failure. Such complications often increase the hospital stay and the cost of treatment; however, the frequency of these complications is comparable to the open technique.[28]

## COMPARISON OF OPEN AND LAPAROSCOPIC REPAIR

The laparoscopic ventral hernia repair has advantages over the open hernia repair in that it has fewer perioperative complications, a reduced hospital stay and fewer hernia recurrences.

One of the greatest benefits of the laparoscopic approach to incisional hernias is the reduction in complications related to wound and mesh infection. The open technique of ventral hernia repair has historically been associated with a high rate of cellulites and mesh infection. In his landmark article, Dr. Stoppa experienced a ‘wound sepsis’ rate of 12%.[11] Wound problems are not unexpected, due to the large soft tissue dissection required for retromuscular placement of large pieces of mesh. Placing the mesh intra-abdominally through a trocar, however, obviates the need for extensive tissue dissection that potentially devascularizes the fascia and causes hematoma formation, both of which contribute to wound and mesh complications. A mesh infection rate of 0.6% and cellulitis of the trocar sites that resolved with antibiotics is seen in 1.1% of cases after laparoscopic repair; these percentages compare favorably with the 12 to 18% rate of wound infection reported in open prosthetic repair series.[14,28]

Only two comparative studies comparing laparoscopic and open ventral hernia repair have been published in the literature. These studies, conducted by Carbajo *et al*[21] and DeMaria *et al,*[29] support the advantages purported by the literature. Based on the data from the comparative studies, postoperative complications are less in the laparoscopic group (23.2 versus 30.2%). Also, the incidence of wound and mesh infections is lower in the laparoscopic patients. Longer operative times and costs have been reported in all comparative series; however, operative times tend to shorten once the plateau of the learning curve for this procedure is reached.

It is however difficult now to conduct a prospective randomized controlled trial comparing the laparoscopic and open approach because strong evidence has emerged in favor of the laparoscopic approach and such a trial may be unethical.

## COST OUTCOMES

There are encouraging results being reported in comparative studies regarding the cost analysis of laparoscopic versus open repair of ventral hernias. In a recent series, laparoscopic ventral hernia repair using a dual-layer polypropylene mesh and transfascial sutures significantly reduced surgical site infections, length of hospital stay and costs as compared to open mesh repair. This study has also shown decreased overall hospital costs for laparoscopic hernia repair despite higher operative costs.[30] However, types of mesh used and fixation device can make sizeable differences in cost calculations. The long-term benefits in terms of early return to work and decreased recurrence rates with laparoscopic repair should also be taken into consideration when deciding on the cost-benefit ratio of laparoscopic versus open repair of ventral hernias.

## CONCLUSION

Laparoscopic repair of ventral / incisional hernias is safe, feasible and effective, with low recurrence rates, low postoperative morbidity and low rates of wound and mesh infection. Operative costs may be optimized with judicious selection of mesh and optimal use of transabdominal sutures and fixation device.
